# Preparation of Mn^2+^ Doped Piperazine Phosphate as a Char-Forming Agent for Improving the Fire Safety of Polypropylene/Ammonium Polyphosphate Composites

**DOI:** 10.3390/ma14247589

**Published:** 2021-12-10

**Authors:** Fuqiang Dong, Zhonglin Luo, Biaobing Wang

**Affiliations:** Jiangsu Key Laboratory of Environmentally Friendly Polymeric Materials, Jiangsu Collaborative Innovation Center of Photovoltaic Science and Engineering, School of Materials Science and Engineering, Changzhou University, Changzhou 213164, China; 19085204286@smail.cczu.edu.cn (F.D.); zhonglinluo@cczu.edu.cn (Z.L.)

**Keywords:** manganese ion, char-forming agent, polypropylene, ammonium polyphosphate, fire safety

## Abstract

A piperazine phosphate doped with Mn^2+^ (HP-Mn), as a new char-forming agent for intumescent flame retardant systems (IFR), was designed and synthesized using 1-hydroxy ethylidene-1,1-diphosphonic acid, piperazine, and manganese acetate tetrahydrate as raw materials. The effect of HP-Mn and ammonium polyphosphate (APP) on the fire safety and thermal stability of polypropylene (PP) was investigated. The results showed that the combined incorporation of 25 wt.% APP/HP-Mn at a ratio of 1:1 endowed the flame retardant PP (PP6) composite with the limiting oxygen index (LOI) of 30.7% and UL-94 V-0 rating. In comparison with the pure PP, the peak heat release rate (PHRR), the total heat release (THR), and the smoke production rate (PSPR) of the PP6 were reduced by 74%, 30%, and 70%, respectively. SEM and Raman analysis of the char residues demonstrated that the Mn^2+^ displayed a catalytic cross-linking charring ability to form a continuous and compact carbon layer with a high degree of graphitization, which can effectively improve the flame retardancy of PP/APP composites. A possible flame-retardant mechanism was proposed to reveal the synergistic effect between APP and HP-Mn.

## 1. Introduction

Polypropylene (PP), as a kind of commodity thermoplastic, has been widely used in many fields, such as the packaging, automotive, and building industries, for its excellent processability, low density, low cost, and outstanding mechanical properties [1,2,3]. Unfortunately, pure PP is readily combustible due to its aliphatic hydrocarbon structure [4,5]. Moreover, extensive smoke and heat are released during the combustion, accompanying severe melt dripping. As such, its extended application is seriously restricted in some fields requiring harsh flame retardancy [6,7]. To date, many efforts have been devoted to improving the flame retardancy of PP [8,9,10,11]. One of the most common ways is to use intumescent flame retardants (IFRs) due to their environment-friendliness and low smoke [12,13]. Traditional IFRs usually consist of three components: acid source, charring agent, and gas source Ref. [14]. However, the traditional IFRs are poorly compatible with non-polar PP substrates and difficult to disperse uniformly, resulting in a low flame retardant efficiency [15,16]. Many works have demonstrated that the UL-94 V-0 rating achieved until the IFR loading level is up to 30 wt.% or above [17,18] Therefore, many approaches have been tried to improve the flame retardant efficiency of the traditional IFRs, including modification of ammonium polyphosphate (APP) [19], preparation of novel charring agent [18], and introduction of synergistic agent [20] etc. Undoubtedly, the synthesis of a superior alternative carbon source is one of the effective ways to improve the flame retardant efficiency of IFR systems.

Hydroxy ethylidene-1,1-diphosphonic acid (HEDP) is a promising acid source with P-C-P structure [21]. Feng et al. [22] found that the compounding of HEDP and ammonium sulfamate (AMS) significantly improved the flame retardant performance and anti-drip of PET. Xia et al. [23] synthesized 1-hydroxy ethylidene-1,1-diphosphonic ammonium (HEDPA), and the results showed that polystyrene/IFR composite achieved UL-94 V-0 rating at the 25 wt.% loading of HEDPA-PER (pentaerythritol)-MEL (melamine) systems. Furthermore, some works have proven that metal ions can effectively improve the charring ability of IFRs [24,25]. For instance, Zhao et al. [26] prepared a three-dimensional flower-like nickel cobaltate (NiCo_2_O_4_) and used it as a synergistic agent for IFR. The PP composite containing 1.5 wt.% NiCo_2_O_4_ and 18.5 wt.% IFR passed the UL-94 V-0 rating test. In the work of Li et al. [27], melamine phytate supramolecular nanosheet doped with Mn^2+^ (PAMA-Mn) was prepared via a hydrothermal procedure. The experimental results showed that the PP composite achieved UL-94 V-0 rating with an LOI value of 31.9% at the incorporation of 4.5 wt.% PAMA-Mn, 9 wt.% APP, and 4.5 wt.% PER. The peak heat release rate and peak smoke production rate of PP composite were reduced by 56% and 23% as compared with the pure PP, respectively. They contributed to the reduction of the fire hazard risk to the in-situ and targeted catalytic cross-linking charring ability of Mn^2+^. Zhang et al. [28] further found that Mn^2+^ has a more highly synergistic effect than other metal ions (Co^2+^, Ni^2+^, Zn^2+^) in PP/APP/PER flame retardant systems.

Given the two geminal phosphonate groups of HEDP, it is feasible to use HEDP as a powerful chelator for complexing metal ions [29]. In the present work, a Mn^2+^-doped piperazine phosphate (HP-Mn) containing a HEDP moiety was designed and prepared through an ionic reaction. It is expected that the HP-Mn acts as a synergistic char-forming agent for PP/APP composites. The flame retardancy and thermal stability of the PP/IFRs composites were investigated, and the synergistic flame retardant mechanism between HP-Mn and APP was elucidated on the basis of the gaseous pyrolysis products and the char residues after a cone calorimetry test (CCT).

## 2. Experimental

### 2.1. Materials

PP (F401, MFR: 2.5 g/10 min) was provided by Yangzi Petroleum Chemical Company (Nanjing, China). Piperazine (AR, waterless) and manganese (II) acetate tetrahydrate (MnAc•4H_2_O, AR) were purchased from Sinopharm Group Co., Ltd. (Shanghai, China). Hydroxy ethylidene-1,1-diphosphonic acid (HEDP crystalline powder, purity > 95%) was supplied by Shandong Taihe Water Treatment Co., Ltd. (Shandong, China). APP (TY432, APP crystalline form II, polymerization degree > 1000) was purchased from Yunnan Tianyao Chemical Co., Ltd. (Kunming, China). All of the marketing materials were used directly without further purification.

### 2.2. Preparation of Mn^2+^-Doped Piperazine Phosphate (HP-Mn)

**Figure 1:**
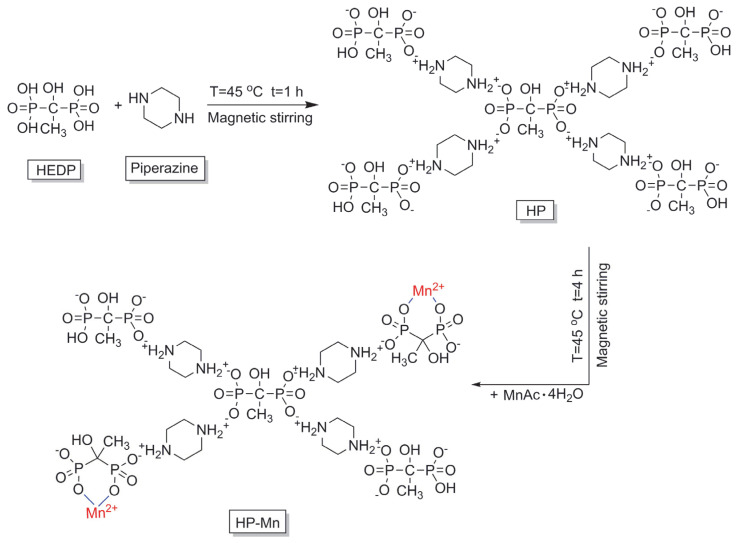
The synthetic route of HP-Mn.

Mn^2+^-doped piperazine phosphate, named as (HP-Mn), was prepared in two steps according to the synthetic route as shown in Figure 1. The first step was to prepare the piperazine phosphate salt through an ionic reaction. To a 250 mL three-necked flask (China National Medicines Co., Ltd. Shanghai, China) equipped with magnetic stirring and a reflux condenser, piperazine (8.64 g, 0.1 mol) and 100 mL deionized water were added and stirred at 45 °C until the piperazine was dissolved completely. Subsequently, HEDP (20.6 g, 0.1 mol) was added to the above solution. Afterwards, the mixture was refluxed at 45 °C for 1 h. Finally, the reaction solution was cooled to room temperature, and solid powder was obtained after vacuum filtration. The product was washed with anhydrous ethanol several times, then vacuum-dried to constant weight at 100 °C and called HP (product yield: 91.3%).

The second step was to prepare HP-Mn through the strong complexation between the phosphate structure in the HP and Mn^2+^. To a 250 mL three-necked flask, HP salt (30.0 g) and 100 mL deionized water were added and magnetically stirred at 45 °C until a clear solution was obtained. Subsequently, 30 mL of MnAc aqueous solution (1.67 mol/L) was added dropwise to the aforementioned solution. Thereafter, the mixture was stirred for another 4 h at 45 °C. During the reaction, it was observed that white solids precipitated slowly. Afterwards, the crude product was cooled to room temperature, collected after filtration, and washed with absolute ethanol and deionized water several times. Finally, the white powder was dried in a vacuum oven (Gongyi Yuhua Instrument Co., Ltd. Henan, China) at 100 °C to a constant weight (product yield: 81.5%).

### 2.3. Sample Preparation

The PP, APP, and HP-Mn were dried in a vacuum oven at 90 °C overnight before using, and the specific formula is listed in Table 1. All PP/IFR composites were prepared on a twin-roller internal mixer (US-70C, Changzhou Suyan Technology Co., Ltd., Changzhou, China) with a roll speed of 60 rpm at 185 °C for 10 min. The PP composites were hot pressed under a plate vulcanizer (ZHY-W, Chengde Testing Machine Factory, Chengde, China) into plates for cone calorimetry, UL-94, and LOI tests.

### 2.4. Characterization

Fourier transform infrared (FTIR) spectra were obtained using a Perkin Elmer instrument (Wikipedia, Waltham, MA, USA). The powders were mixed with KBr pellets, and the wavenumber range was set from 4000 to 450 cm^−1^.

An X-ray diffraction (XRD) test was performed on a power D/MAX-2500 diffraction (Rigaku Corporation, Tokyo, Japan) using Cu-Kα radiation with a scanning 2θ angle ranging from 5° to 50°.

X-ray photoelectron spectroscopy (XPS) was collected on an ESCALAB 250XI system (Thermo Fischer, Waltham, MA, USA) using Al-Kα radiation (hn = 1486.6 eV).

The UL-94 vertical burning test was performed on a CZF-5 instrument (Shine Ray Instrument Co. Ltd., Nanjing, China) according to ASTMD3801 with the specimens in 130 mm × 13 mm × 3 mm. Limiting oxygen index (LOI) was carried out on an LOI analyzer (JF-3, Jiang Ning Co. Ltd., Nanjing, China) according to GB/T 2406-93 standard and the specimen dimension used for test was 130 mm × 6.5 mm × 3 mm. Both the burning time and the LOI values were obtained from the average value of five replicates.

Thermogravimetric analysis (TGA) was recorded on a Perkin-Elmer TGA 4000 (Wikipedia, Waltham, MA, USA). Approximately 10 mg of the sample was placed in an alumina crucible and heated from 30 to 800 °C at a heating rate of 10 °C/min under nitrogen and oxygen atmosphere flow of 20 mL/min. TG-FTIR analysis was performed under a nitrogen atmosphere at a heating rate of 10 °C/min from 30 °C to 800 °C.

A cone calorimetry test (CCT) was conducted using a cone calorimeter (FTT, East Grinstead, UK) under an external heat flux of 35 kW m^−2^ according to ISO 5660-1, and the dimension of square sample was 100 mm × 100 mm × 3 mm. Three replicates were tested to collect the average data of each point.

Scanning electron microscopy (SEM) with energy dispersive spectroscopy (EDS) was carried out by field emission scanning electron microscope (Zeiss SUPRA55, Jena, Germany) and X-ray energy spectrum (X-Max, Oxford, UK). The microstructures of specimen were observed at an acceleration voltage of 5 kV after gold-spraying and elemental analysis.

Raman spectroscopy measurement was measured by a DXR laser Raman spectrometer (Thermo Scientific, Waltham, MA, USA) in the range of 50–3500 cm^−1^.

## 3. Results and Discussion

### 3.1. Characterization of HP and HP-Mn

**Figure 2:**
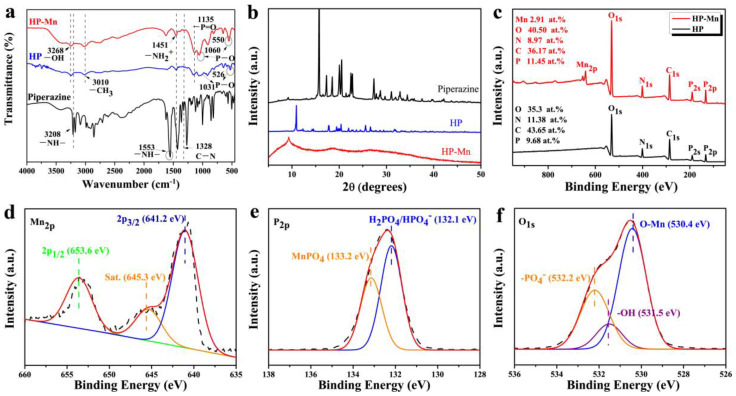
FTIR spectra (**a**) and XRD patterns (**b**) of piperazine, HP and HP-Mn; XPS survey spectra of HP-Mn and HP (**c**); high-resolution XPS spectra of HP-Mn (**d**–**f**).

Figure 2 presents the FTIR spectra, XRD patterns and XPS diffractions of HP and HP-Mn. As can be seen in Figure 2a, the characteristic absorption peaks of piperazine at 3208 and 1553 cm^−1^ correspond to the stretching and bending vibration of the -NH- groups, respectively, and the one at 1328 cm^−1^ is ascribed to the stretching vibration of the C-N bond [30]. With respect to the FTIR spectrum of HP, the characteristic absorption peaks of HEDP are observed at 3268 cm^−1^ (-OH, stretching vibration), 3010 (-CH_3_, stretching vibration), 1135 (-P=O, stretching vibration), 1031 (P-O, stretching vibration), and 526 cm^−1^ (P-O, bending vibration) [31,32]. Notably, the disappearance of -NH- and the formation of a new peak of -NH_3_^+^ at 1451 cm^−1^ indicate that piperazine reacted with HEDP completely. After doping the Mn^2+^, most of characteristic absorption peaks of HP are also observed in the FTIR spectra of HP-Mn. However, it should be pointed out that the stretching (1031 cm^−1^) and bending (526 cm^−1^) vibration absorption peaks of P-O are blue shifted to 1060 and 550 cm^−1^, respectively, indicating that HP interacts with Mn^2+^ through the P-O- group in HEDP [27,33].

As illustrated in Figure 2b, the XRD pattern of piperazine displays many sharp peaks over the range of 15–30°, indicating its complex crystalline structure. By contrast, the characteristic diffraction peak at approximately 15.6° disappears, but a new diffraction peak at 11° occurs in the XRD pattern of HP, whic indicates that the crystalline structure of HP is different from the piperazine. With respect to the XRD pattern of HP-Mn, no discernible diffraction peaks of HP are detected except one new diffraction peak at approximately 9.2°, which indicates that the crystalline structure of HP becomes semicrystalline after doping with the Mn^2+^. Obviously, the results confirm that the Mn^2+^ is successfully doped into HP. XPS spectra were adapted to analyze the elemental composition and chemical valence of HP and HP-Mn, as displayed in Figure 2c–f. In the full survey of HP and HP-Mn (Figure 2c), characteristic peaks at 530.7, 401.2, 285.6, and 132.5 eV are attributed to O 1s, N 1s, C 1s, and P 2p, respectively. By comparison with the HP, a new peak at 641.2 eV (Mn 2p) is clearly identified in the spectrum of HP-Mn, demonstrating that the Mn^2+^ was successfully doped into HP. In the Mn 2p high resolution spectrum of HP-Mn (Figure 2d), the peak can be deconvoluted into three peaks at 641.2 eV (Mn 2p_3/2_), 653.6 eV (Mn 2p_1/2_, manganese(II) phosphate), and 645.3 eV (satellite peak of Mn 2p_3/2_) [34,35]. The peaks of P 2p high resolution spectrum (Figure 2e) at 132.1 eV and 133.2 eV correspond to manganese phosphate and phosphoric acid/hydrogen phosphate [36,37]. In the O 1s high resolution spectrum (Figure 2f), the peak at 532.2 eV is ascribed to phosphate, while the signals at 531.5 eV and 530.4 eV are attributed to the -OH and O-Mn, respectively [38]. As such, it can be concluded that Mn^2+^ is successfully doped into HP by complexation.

**Figure 3:**
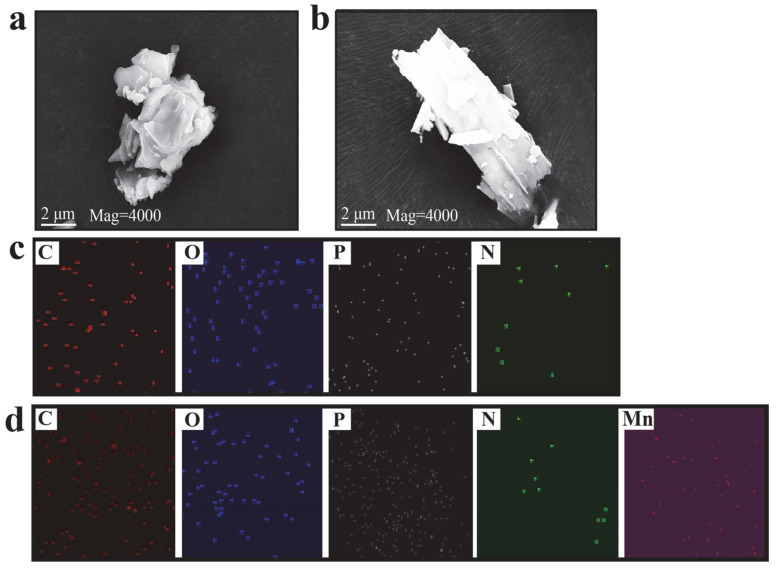
SEM images of HP (**a**) and HP-Mn (**b**); EDS mapping of HP (**c**) and HP-Mn (**d**).

The SEM images with EDS mapping of HP and HP-Mn are illustrated in Figure 3. In contrast to the irregular shape of HP (Figure 3a), the HP-Mn presents a flake structure (Figure 3b). Such changes in the morphology may result from the strong chelation between Mn^2+^ and the phosphoric group, which was replaced partially by the hydrogen bond between piperazine and HEDP. As can be seen in the EDS mapping (Figure 3c,d), the nitrogen phosphorus and manganese elements are distributed on HP or HP-Mn uniformly, suggesting that HP and HP-Mn have been successfully prepared.

### 3.2. Flame Retardancy

The flame retardancy of the pure PP and its composites were evaluated by the UL-94 vertical burning test and LOI measurement, and all resultant data are listed in Table 2. Clearly, the pure PP gives a low LOI value of 18.5% and no UL-94 rating is obtained. With the incorporation of 25 wt.% flame retardant (APP, HP, or HP-Mn (PP3) alone, there is still no UL-94 rating achieved although the LOI values of the flame retardant composites increase. This indicates that the flame retardant effect is not enough in the case of the usage of the acid source or carbon source alone. However, the flame retardancy is improved with the incorporation of 25 wt.% APP/HP or APP/HP-Mn at a weight ratio of 1:1. For example, the LOI value of PP4 (25 wt.% APP/HP at weight ratio of 1:1) is significantly increased to 27.6% but with a UL-94 V-1 rating. The PP6 with 25 wt.% APP/HP at a weight ratio of 1:1 gives a high LOI value of 30.7% and achieves the UL-94 V-0 rating. The improved IFR effect of HP-Mn is attributed to the catalytic cross-linking charring ability of Mn^2+^ [39,40]. The results indicate that HP-Mn works better in coordination with APP to exert a synergistic flame-retardant effect on PP.

**Figure 4:**
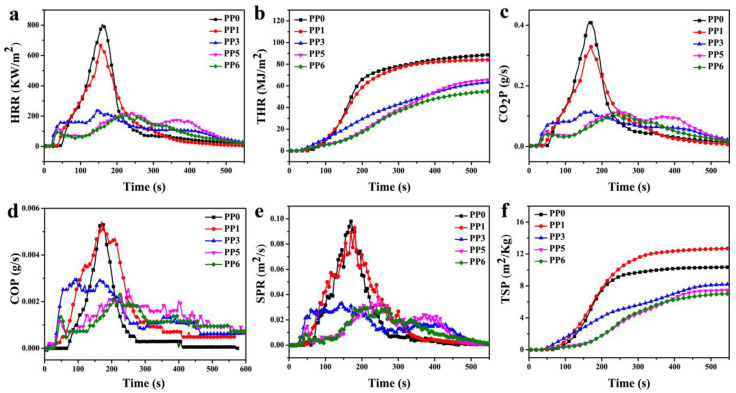
HRR (**a**), THR (**b**), CO_2_P (**c**), COP (**d**), SPR (**e**) and TSP (**f**) curves of samples during the combustion in CCT.

The cone calorimetry test (CCT) was used to investigate the combustion behaviors of the pure PP and its flame-retardant composites, and five typical samples of PP0, PP1, PP3, PP5, and PP6 were selected. Some representative results are shown in Figure 4 and Table 3.

As compared with the pure PP, the TTI (the time to ignition) values of all flame retardant composites are decreased, which should be due to the earlier decomposition of PP caused by the flame retardants. Generally, the heat release rate (HRR) and total heat release (PHHR) curves reveal the propensity of fire growth rate and flame spread during fire accidents. After ignition, the pure PP burned violently, accompanying severe melt-dripping during CCT, reaching the peak heat release rate (PHRR) of 804.3 kW/m^2^ at 160 s with THR value of 88.8 MJ/m^2^. With the addition of 25 wt.% APP (PP1) or HP-Mn (PP3) alone, the PHRR values are reduced to 667.0 and 242.5 kW/m^2^, respectively. It is worth noting that the PP6 sample gives the lowest PHRR and THR values, with a reduction of 74% and 30% as compared with the neat PP, respectively. In addition, the HRR curve (Figure 3a) of the PP/APP/HP-Mn composite (PP6) presents several peaks in the rate of heat release, which is a typical behavior of highly efficient IFR systems [41].

Since large amounts of smoke released from the burning of polymer materials cause asphyxiation, the smoke suppression capability is also very crucial for the flame retardants. As shown in Figure 4c,d, the CO_2_ production rate (CO_2_P) and CO production rate (COP) values are significantly decreased with the addition of 25 wt.% APP/HP-Mn (1:1), that is, the mean yield of CO_2_ (CO_2_Y) is decreased from 2.49 kg/kg for neat PP to 2.09 kg/kg for PP6 by 16.1% reduction. This phenomenon is attributed to the APP/HP-Mn flame retardant systems that could form a stable carbon layer, inhibiting the progress of combustion. However, the mean yield of CO (COY) of all composites is significantly increased in comparison with the pure PP, which is attributed to the incomplete combustion of PP in the presence of APP and HP-Mn. With respect to the peak smoke production rate (PSPR) and total smoke production (TSP), the PP/APP composite gives an almost unchanged PSPR value but a higher TSP value as compared with the pure PP, which is due to the incomplete combustion of PP in the presence of APP [42]. For the PP/APP/HP-Mn composite (PP6), the lowest PSPR (0.029 m^2^/s) and TSP (7.2 m^2^) values are achieved, with a reduction of 70% and 30% in comparison with the pure PP, respectively. Hereby, it is evident that the APP/HP-Mn system has an efficient smoke suppression ability.

Finally, the flame retardant index (FRI) and fire growth index (FGI) are used to assess the flame retarded performance of thermoplastic composites based on CCT data [43,44]. The FRI and FGI are obtained by the following equations.

$$FRI=\frac{{\left[THR\times \left(\frac{PHRR}{TTI}\right)\right]}_{Pure\;PP}}{{\left[THR\times \left(\frac{PHRR}{TTI}\right)\right]}_{PP\;composites}}\;FGI=PHRR/T_{PHRR}$$

Generally, the higher FRI and lower FGI values mean that the material has better fire safety, and the FRI and FGI values of the pure PP and its composites are listed in Table 2. Obviously, the PP6 sample with 25 wt.% APP/HP-Mn (1:1) gives the highest FRI value (3.34) but the lowest FGI value (0.92 kW/m^2^s). This indicates that APP and HP-Mn synergistically improve the fire safety of the flame retardant composites.

### 3.3. Analysis of Thermal Stability

**Figure 5:**
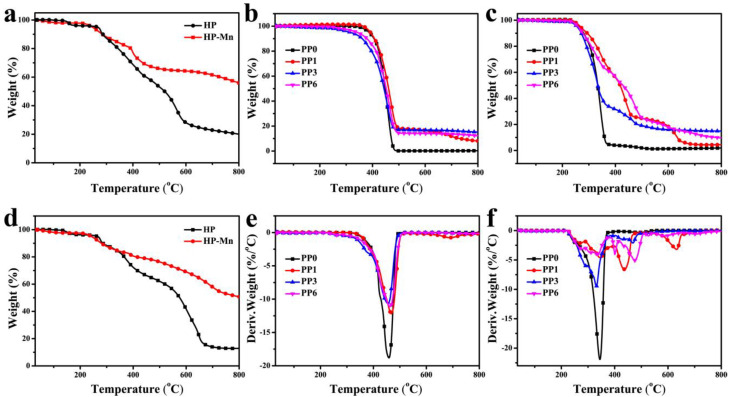
TGA (**a**–**d**) and DTG (**e**,**f**) of flame retardant, pure PP and flame retardant composites under nitrogen (**a**,**b**,**e**) and air atmosphere (**c**,**d**,**f**).

TG and DTG curves shown in Figure 5 are used to investigate the thermal degradation behaviors of the flame retardants (HP and HP-Mn), pure PP, and its flame-retardant composites under nitrogen atmosphere and air atmosphere. The resultant data are listed in Table 4 and Table 5, respectively.

As can be seen in Figure 5a, both HP and HP-Mn present good thermal stability under nitrogen atmosphere and the initial decomposition temperatures (T_5 wt.%_, defined as the temperature at which 5.0 wt.% mass loss occurred) are 267.2 °C and 247.0 °C, respectively, which meet the processing temperature of PP. Additionally, although the HP-Mn decomposes early than HP, its residual yield (55.8%) is far greater than the later (20.0%). This indicates that the HP-Mn possesses excellent char-forming ability than HP. This may contribute to the fact that Mn^2+^ can catalyze the earlier thermal decomposition of HP and promote it to form char with high quality, which acts as a physical barrier to protect the further degradation of the inward matrix. Similar thermal degradation behavior is observed for HP and HP-Mn under air atmosphere (Figure 5d).

As shown in the TGA (Figure 5b) and DTG (Figure 5e) curves of the pure PP and its flame-retardant composites under nitrogen atmosphere, the pure PP (PP0) presents a single degradation stage, giving T_5 wt.%_ of 387.2 °C and T_max1_ (maximum mass loss rate temperature) of 458.0 °C, and no residue is left at 800 °C. For PP1 containing 25 wt.% APP alone, its T_5 wt.%_ and T_max_ values are slightly increased due to the high stability of APP Ref. [45], with a residual yield of 8.0%. Conversely, the PP3 containing 25 wt.% HP-Mn alone gives the lowest T_5 wt.%_ and T_max_ values and the highest residual yield of 15.1 wt.% due to the lower T_5 wt.%_ of HP-Mn and the catalytic carbonization effect of Mn^2+^. Taking the comprehensive effect of APP and HP-Mn into consideration, there is no doubt that the PP6 containing 25 wt.% APP/HP-Mn gives T_5 wt.%_ of 327.3 °C, T_max_ of 467.7 °C, and residual yield of 12.3 wt.%. Furthermore, it is noteworthy that the degradation rate (Rmax) of the flame retardant composites containing HP-Mn (PP3 and PP6) are lower than that of PP0 or PP1, indicating that the presence of HP-Mn can inhibit further decomposition due to the catalytic carbonization effect of Mn^2+^.

With respect to the thermal degradation behavior under air atmosphere (Figure 5c,f), the flame-retardant composites exhibit a multi-stage decomposition process due to the thermal oxidation chemical reaction. Furthermore, the flame retardant PP composites also show lower T_5%_ and Rmax values than that of PP in air. This means that the flame retardants promote not only the decomposition process of PP at low temperature, but also the formation of more char. The char can hinder the further combustion of the inward matrix, and the R_max_ is thus reduced.

### 3.4. Flame-Retardant Mechanism Analysis

#### 3.4.1. Gas Phase Analysis

**Figure 6:**
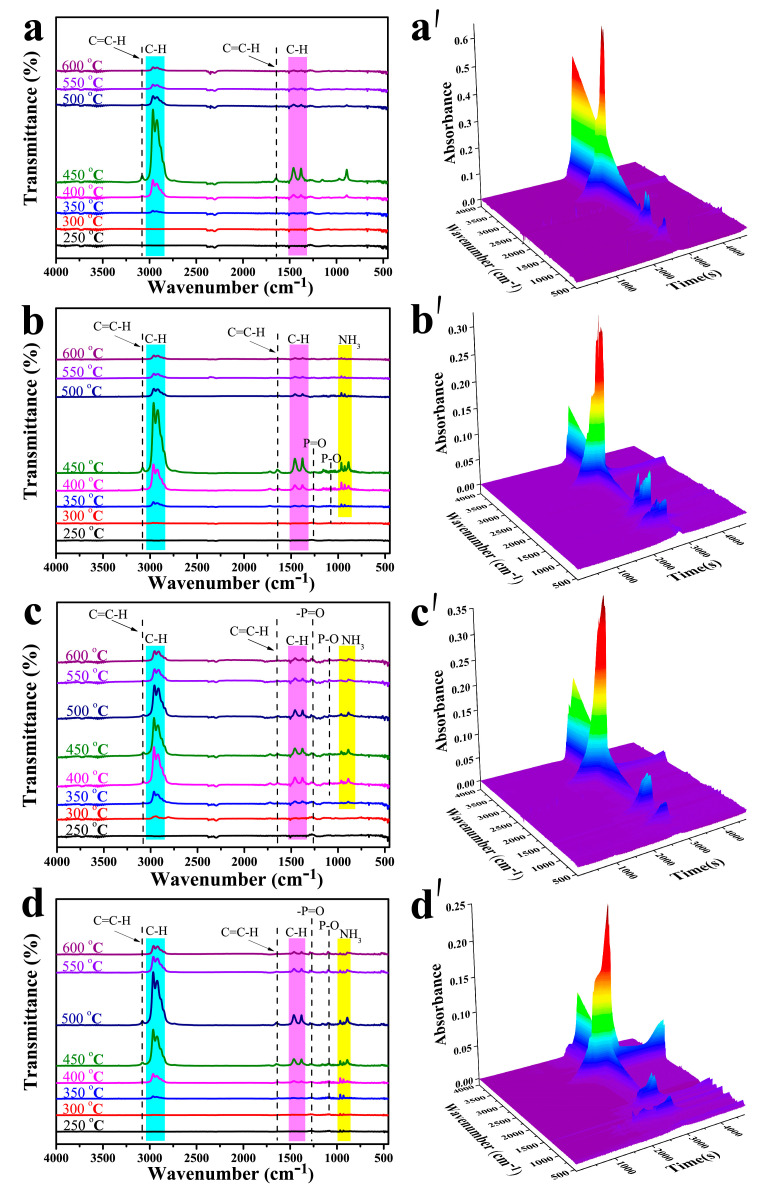
FTIR spectra and 3D TG-FTIR of the pyrolysis products of PP0 (**a**,**a′**), PP1(**b**,**b′**), PP3 (**c**,**c′**) and PP6 (**d**,**d′**) at different temperatures.

TG-FTIR was used to analyze the gaseous products during the thermal degradation process of the pure PP and its composites. The 3D TG-FTIR spectra and the characteristic spectra of the gaseous pyrolysis products obtained at different temperatures are shown in Figure 6. For the pure PP (PP0), the flammable gaseous pyrolysis products are all attributed to hydrocarbons, including alkene (3077 and 1645 cm^−1^), hydrocarbons (2957 and 2918 cm^−1^), and diene (1378 cm^−1^) [46]. With respect to the flame retardant composites, new characteristic peaks at 1278 and 1092 cm^−1^ are attributed to P=O and P-O, which are produced from the decomposition of polyphosphoric acids and APP [47]. In addition, the appearance of characteristic absorption peaks at 927 and 968 cm^−1^ reveals the release of NH_3_ from the degradation of APP and the piperazine ring [48].

#### 3.4.2. Analysis of Char Residue

**Figure 7:**
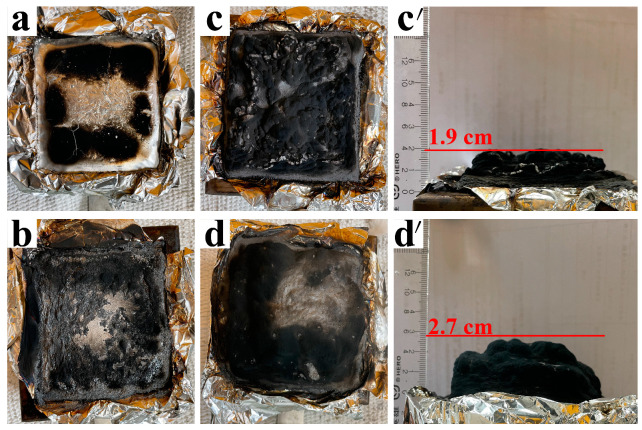
Digital photographs of the char residue of PP0 (**a**), PP1 (**b**), PP3 (**c**,**c**′), and PP6 (**d**,**d**′) after CCT.

**Figure 8:**
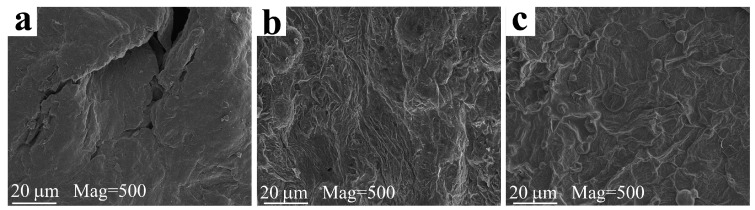
SEM images of the char residue obtained after CCT for PP1 (**a**), PP3 (**b**), PP6 (**c**).

Figure 7 shows digital photographs of the char residues after the CCT. Clearly, there is almost no residue for pure PP (Figure 7a), and a trace of residues is left for the PP1 containing APP alone (Figure 7b). At loading of HP-Mn alone, an expanded char residue with height of 1.9 cm is obtained. This indicates that the HP-Mn has superior char-forming ability over the APP. Notably, with the combined incorporation of APP and HP-Mn, much intumescent and denser carbon layer with expansion height of 2.7 cm are achieved after CCT. This suggests that there is a synergistic effect between APP and HP-Mn on the formation of intumescent char layer, which can act as physical barrier to interrupt the transition of the heat and gases. The micromorphology of the char residue was further revealed via SEM images, as shown in Figure 8. Obviously, some large cracks are presented in the char residue of the PP1 specimen containing APP alone (Figure 8a). Although a thicker and denser char layer is achieved for the PP3 specimen containing HP-Mn alone, there are still some visible microvoids (Figure 8b). Furthermore, with the combined incorporation of APP and HP-Mn, the char residues of PP6 sample display a continuous and compact surface without visible microvoids (Figure 8c), which can prevent further combustion of the underlying polymer matrix.

**Figure 9:**
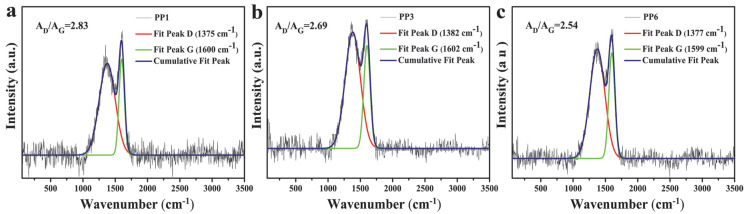
Raman spectra of char residues of PP1 (**a**), PP3 (**b**), PP6 (**c**) after CCT.

The quality of char residues after CCT was further characterized via Raman spectroscopy, as depicted in Figure 9. Two sharp peaks at 1377 cm^−1^ and 1600 cm^−1^ are attributed to the D and G bands, representing the graphitic structure and lattice defect of carbon atom, respectively. Generally, the area ratio (A_D_/A_G_) of D and G bands reveals the graphitization degree of the char residue, and a lower A_D_/A_G_ value means a higher graphitization degree and better quality of the char layer [49]. The calculated A_D_/A_G_ values are in descending order of PP1 (2.83) > PP3 (2.69) > PP6 (2.54), which indicates that the combination of APP and HP-Mn can improve synergistically the quality of char residue as compared with the incorporation of APP or HP-Mn alone, which facilitates the formation of much denser and continuous char layers with high strength. The flame retardancy, consequently, is improved with the combined incorporation of APP and HP-Mn.

**Figure 10:**
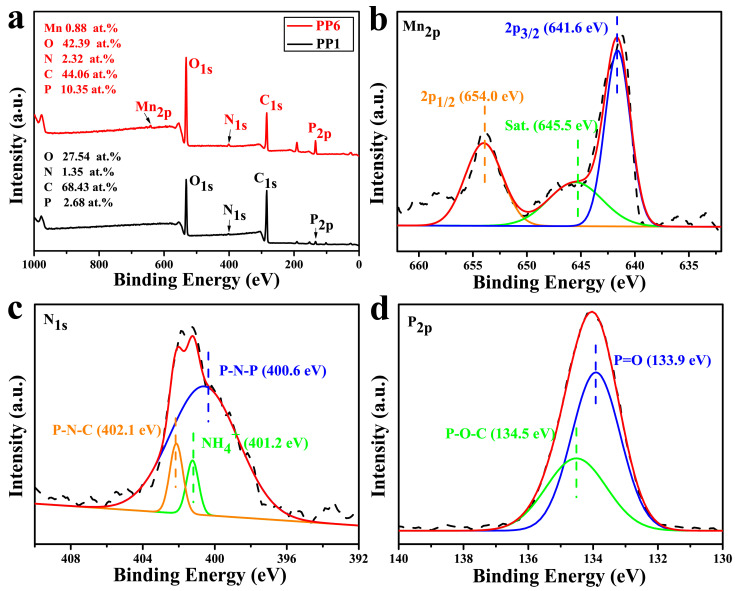
XPS survey spectra of PP1 and PP6 (**a**); high-resolution XPS spectras of PP6 (**b**–**d**).

Furthermore, the elemental compositions of the char residue of PP1 and PP6 were measured by XPS analysis, and the corresponding spectra with Mn 2p, N 1s, and P 2p high-resolution are shown in Figure 10. Obviously, four common elements, including O, N, C, and P, are found in the residue of both PP1 and PP6 (Figure 10a). In comparison with PP1, a new peak at 641.4 eV (Mn 2p) appears in the spectra of char residue of PP6. Moreover, there are higher O and P contents in the char residues of PP6 sample as compared with PP3. It might be due to that the catalytic cross-linking ability of Mn^2+^ promotes the formation of more phosphorus-oxygen bonds in the char residue during combustion. For Mn 2p spectrum (Figure 10b), the peaks at 641.6 eV, 645.5 eV, and 654.0 eV are assigned to Mn 2p_3/2_, the satellite peaks of Mn 2p_3/2_ and Mn 2p_1/2_, respectively. In N 1s spectrum (Figure 10c), the peaks at 400.6 eV, 401.2 eV, and 402.1 eV are attributed to P-N-P, NH_4_^+^, and P-C-N, respectively [50]. For P 2p spectrum (Figure 10d), the peaks at 133.9 eV and 134.5 eV correspond to P=O and P-O-C groups in polyphosphoric acid and pyrophosphoric acid [51], respectively. All XPS results show that the Mn^2+^ plays an indispensable role in the generation of high-quality carbon layers and the APP/HP-MN system can promote the formation of cross-linking structures during combustion, which facilitates the formation of a high-quality char layer.

#### 3.4.3. Possible Flame-Retardant Mechanism

**Figure 11:**
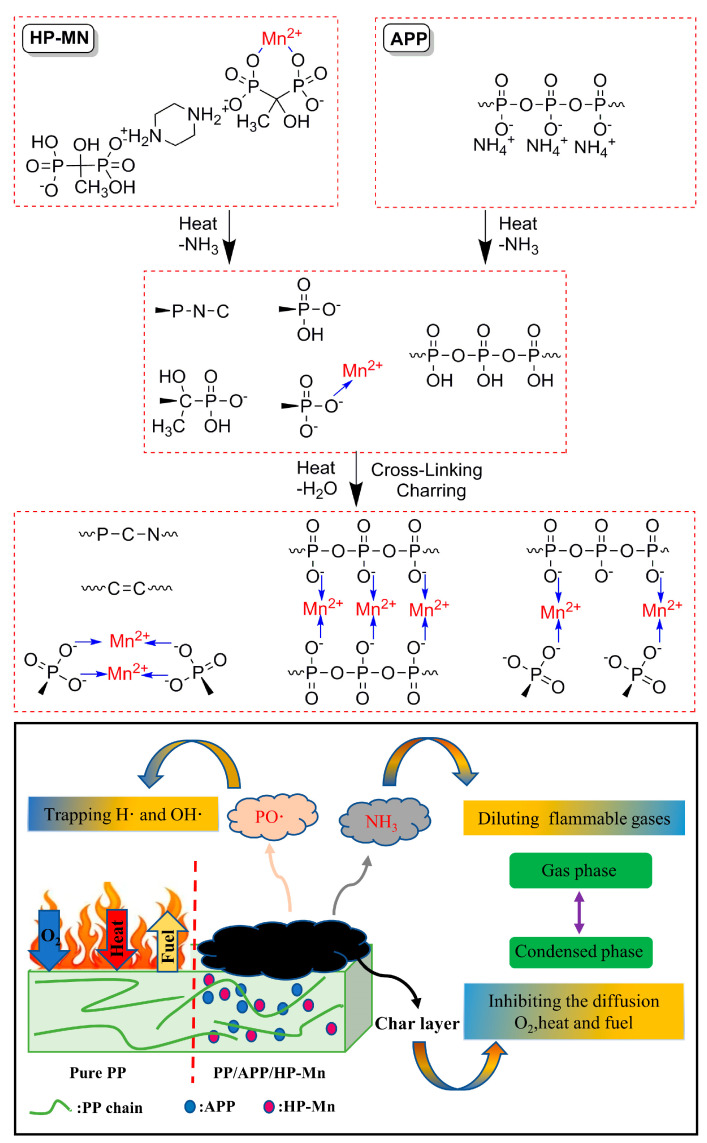
Schematic diagram of the flame-retardant mechanism.

Based on the above analysis of the gaseous pyrolysis products and the char residues after CCT, the flame retardant mechanism of PP/APP/HP-Mn composites is illustrated in Figure 11. In the gas phase, the decomposition of HP-Mn and APP produces not only non-combustible gases (NH_3_), but PO• radicals and Mn^2+^ which can trap the free radicals produced by PP during the combustion. In the condensed phase, P-N-C groups that acted as carbon source reacted with other products decomposed by HP-Mn in the initial stage of the combustion and formed a high-quality char layer at a higher temperature under the catalysis of APP. A high-quality char layer could act as a barrier to prevent further decomposition of the PP composites. It is worth noting that Mn^2+^ can not only bridge adjacent -PO^−^, but also HP-Mn and APP, and then form a compact and strong char layer in a short time.

## 4. Conclusions

In summary, a novel charring agent doped with Mn^2+^ (HP-Mn) was successfully prepared, and its structure was characterized by FTIR, XRD, and XPS, respectively. The combination of APP and HP-Mn shows better flame retardancy than the usage of APP or HP-Mn alone. An LOI value of 30.7% and UL-94 V-0 rating were achieved for the PP composite with incorporation of 25 wt.% APP/HP-MN at a ratio of 1:1. In contrast with the neat PP, the PHRR, TSP, and PSPR were also decreased by 74%, 30%, and 70%, respectively. The decomposition of HP-Mn and APP produces not only non-combustible gases (NH_3_), but PO• radicals and Mn^2+^ in the gas phase, which can trap the free radicals produced during the combustion of PP. Furthermore, a continuous and compact carbon layer with high degree of graphitization acted as a physical barrier to hinder the transfer of the heat and flammable substances in the condensed phase. This demonstrated that the HP-Mn was an efficient charring agent in the IFR system for the flame retardant PP.

## Figures and Tables

**Table 1:** The specific formulation of the PP/IFR composites.

Sample ID	PP, wt.%	APP, wt.%	HP, wt.%	HP-Mn
PP0	100	/	/	/
PP1	75	25	/	/
PP2	75	/	25	/
PP3	75	/	/	25
PP4	75	12.5	12.5	/
PP5	77.5	11.25	/	11.25
PP6	75	12.5	/	12.5

**Table 2:** Detailed results for PP and its flame-retardant composites from UL-94 and LOI tests.

Sample ID	LOI (%)	UL-94 Rating (3 mm)	t_1_/t_2_	Dropping or Not
PP0	18.5 ± 0.3	NO rate	>30	Yes
PP1	20.1 ± 0.2	NO rate	>30	Yes
PP2	23.5 ± 0.4	NO rate	>30	Yes
PP3	25.3 ± 0.3	NO rate	>30	Yes
PP4	27.6 ± 0.2	V-1	2.2/9.2	No
PP5	27.2 ± 0.4	V-2	2/14.4	Yes
PP6	30.7 ± 0.2	V-0	1.8/4.6	No

**Table 3:** Data of the pure PP and its flame-retardant composites during cone calorimeter combustion.

Sample	PP0	PP1	PP3	PP5	PP6
TTI (s)	56 ± 5	45 ± 3	28 ± 1	32 ± 2	31 ± 3
PHRR (kW/m^2^)	804.3 ± 23.5	667.0 ± 15.7	242.5 ± 20.8	226.8 ± 16.2	207.5 ± 10.4
T_PHRR_ (s)	160 ± 5	155 ± 2	150 ± 4	240 ± 2	225 ± 3
THR (MJ/m^2^)	88.8 ± 2.5	83.7 ± 1.8	65.4 ± 2.1	66.4 ± 2.0	57.0 ± 2.3
TSP(m^2^)	10.3 ± 0.5	12.7 ± 0.8	8.4 ± 0.3	7.6 ± 0.4	7.2 ± 0.2
Mean COY (kg/kg)	0.03 ± 0.004	0.04 ± 0.002	0.05 ± 0.003	0.04 ± 0.002	0.06 ± 0.002
PSPR (m^2^/s)	0.098 ± 0.003	0.093 ± 0.005	0.033 ± 0.002	0.038 ± 0.002	0.029 ± 0.003
Mean CO_2_Y (kg/kg)	2.49 ± 0.05	1.90 ± 0.02	2.21 ± 0.09	2.23 ± 0.03	2.09 ± 0.07
Av-EHC (MJ/kg)	39.8 ± 1.2	29.9 ± 0.7	34.2 ± 1.0	34.8 ± 0.5	32.9 ± 0.8
FGI (kW/m^2^s)	5.04	4.30	1.61	0.94	0.92
FRI	1.00	1.02	2.25	2.71	3.34

**Table 4:** TG and DTG curves of HP, HP-Mn, Pure PP and its flame-retardant composites under nitrogen atmosphere.

Sample Code	Nitrogen				
	T_5 wt.%_ (°C)	T_max1_ (°C)	T_max2_ (°C)	Rate of T_max_ ( wt.%/min)	Residual Weight at 800 °C ( wt.%)
HP	267.2	280.0	566.7	3.7	20.0
HP-MN	247.0	273.6	400.7	3.3	55.8
PP0	387.2	458.0	/	18.8	0
PP1	391.6	466.8	/	12.2	8.0
PP3	316.1	456.8	/	10.7	15.1
PP6	327.3	467.7	/	11.1	12.3

**Table 5:** TG and DTG curves of HP, HP-Mn, Pure PP and its flame-retardant composites under air atmosphere.

Sample Code	Air					
	T_5 wt.%_ (°C)	T_max1_ (°C)	T_max2_ (°C)	T_max3_ (°C)	Rate of T_max_ ( wt.%/min)	Residual Weight at 800 °C ( wt.%)
HP	265.9	278.7	606.2	/	3.9	12.8
HP-Mn	242.2	269.2	390.9	698.3	1.8	50.7
PP0	262.8	342.8	/	/	21.5	0
PP1	268.2	344.6	437.5	631.2	6.5	4.5
PP3	255.7	308.7	439.9	/	6.4	14.9
PP6	257.0	279.0	408.5	474.9	5.0	9.7

## Data Availability

Not applicable.
